# Publishing Identifiable Patient Photographs in Scientific Journals: Scoping Review of Policies and Practices

**DOI:** 10.2196/37594

**Published:** 2022-08-31

**Authors:** Marija Roguljić, Dina Šimunović, Tina Poklepović Peričić, Marin Viđak, Ana Utrobičić, Matko Marušić, Ana Marušić

**Affiliations:** 1 Department of Oral Medicine and Periodontology University of Split School of Medicine Split Croatia; 2 Private Dental Practice Split Croatia; 3 Department of Prosthodontics, Study of Dental Medicine School of Medicine, University of Split Library Split Croatia; 4 Department of Research in Biomedicine and Health Center for Evidence-based Medicine University of Split School of Medicine Split Croatia; 5 University of Split Library Split Croatia

**Keywords:** identifiable patient photographs, medical photography, data protection, patient privacy, confidentiality, informed consent, ethical publishing, scientific journals, open access, scoping review, mobile phone

## Abstract

**Background:**

Publishing identifiable patient data in scientific journals may jeopardize patient privacy and confidentiality if best ethical practices are not followed. Current journal practices show considerable diversity in the publication of identifiable patient photographs, and different stakeholders may have different opinions of and practices in publishing patient photographs.

**Objective:**

This scoping review aimed to identify existing evidence and map knowledge gaps in medical research on the policies and practices of publishing identifiable photographs in scientific articles.

**Methods:**

We performed a comprehensive search of the Cochrane Central Register of Controlled Trials, Cochrane Database of Systematic Reviews, CINAHL with Full Text, Database of Abstracts of Reviews of Effects, Ovid MEDLINE, and Scopus. The Open Science Framework, PROSPERO, BASE, Google Scholar, OpenGrey, ClinicalTrials.gov, the Campbell Collaboration Library, and Science.gov were also searched.

**Results:**

After screening the initial 15,949 titles and abstracts, 98 (0.61%) publications were assessed for eligibility at the full-text level, and 30 (0.19%) publications were included in this review. The studies were published between 1994 and 2020; most had a cross-sectional design and were published in journals covering different medical disciplines. We identified 3 main topics. The first included ethical aspects of the use of facial photographs in publications. In different clinical settings, the consent process was not conducted properly, and health professionals did not recognize the importance of obtaining written patient consent for taking and using patient medical photographs. They often considered verbal consent sufficient or even used the photographs without consent. The second topic included studies that investigated the practices and use of medical photography in publishing. Both patients and doctors asked for confidential storage and maintenance of medical photographs. Patients preferred to be photographed by their physicians using an institutional camera and preferred nonidentifiable medical photographs not only for publication but also in general. Conventional methods of deidentification of facial photographs concealing the eye area were recognized as unsuccessful in protecting patient privacy. The third topic emerged from studies investigating medical photography in journal articles. These studies showed great diversity in publishing practices regarding consent for publication of medical photographs. Journal policies regarding the consent process and consent forms were insufficient, and existing ethical professional guidelines were not fully implemented in actual practices. Patients’ photographs from open-access medical journals were found on public web-based platforms.

**Conclusions:**

This scoping review showed a diversity of practices in publishing identifiable patient photographs and an unsatisfactory level of knowledge of this issue among different stakeholders despite existing standards. Emerging issues include the availability of patients’ photographs from open-access journals or preprints in the digital environment. There is a need to improve standards and processes to obtain proper consent to fully protect the privacy of patients in published articles.

## Introduction

### Background

Scientific publications are considered to be the most important formal elements of scholarly communication, translating new evidence in practice and increasing relevant stakeholders’ knowledge. Publishing identifiable patient photographs in journals, such as photographs of the face, is a challenging ethical issue, not only because it requires consent but also because many research participants as well as researchers are not aware of what happens when identifying photographs of individual persons are published. This is of particular importance in digital publishing, especially when open publishing licenses such as the Creative Commons license CC BY are used [1]. Many medical journals that publish articles identifying patient photographs are in open access and under open licenses [2], which means that identifiable patient data are widely available and can be easily abused [3].

It is not always clear how patient data are classified as identifiable, nonidentifiable, or anonymized. The scoping review by Chevrier et al [4] on the use and understanding of terms of anonymization and deidentification in biomedical literature showed that there is large variability in the use of these as well as the need for clearer definitions and better education. Current publication standards on the use of patient identifiable data proposed by the International Committee of Medical Journal Editors (ICMJE) recommend avoiding publication of identifiable photographs or marks and the necessity of obtaining written consent from the patient [5]. However, in some clinical disciplines such as those involving the head and neck, it is necessary to show the patients’ faces to illustrate study findings. The most used methods of deidentification to protect patient identity—covering the eye area in facial photographs—have been recognized as insufficient and should not be published without the patient’s written consent [6-8]. Consent for the publication of an identifiable photograph should be obtained after the patient is informed of all the potential consequences of a publication even if the publication results from routine health care and is written up as a case report [9-12]. Patients should also be aware of the impossibility of withdrawing or controlling any future use of photographs once they have been published on the web [1,13]. This means that consent for the publication of an identifying patient photograph is separate from and additional to the general consent for research [2,8,14-16].

### Objectives

Despite these recommendations, there is diversity among medical journals in their policies on patient consent for the publication of identifying photographs and their implementation in practice [2,17]. There are also varying opinions and practices among different stakeholders—patients, professionals, journals, and professional societies. To identify the existing evidence and map knowledge gaps in research on the policies and practices of publishing identifiable photographs in medicine, we performed a scoping review of the published literature on this topic. The research question of this scoping review was as follows: what are the opinions, standards, and practices of different stakeholders (patients, health professionals, policy makers, journals, editors, and publishers) regarding consent for publishing potentially identifiable medical photographs?

## Methods

### Methodological Approach

We used the methodology for scoping reviews from the Joanna Briggs Institute [18]. The protocol of this scoping review was registered at the Open Science Framework [19]. Study results are presented following the PRISMA-ScR (Preferred Reporting Items for Systematic Reviews and Meta-Analyses extension for Scoping Reviews) checklist (Multimedia Appendix 1) [20].

### Study Selection (Eligibility Criteria)

We performed a sensitive search without language, time, or geographical limitations to identify studies that investigated the conditions of publication of patient facial photographs regardless of whether they were identifiable or not; articles that addressed only body parts other than the face were excluded. Publications that reported the results of conducted studies were included in the analysis. All other types of publications, such as editorials, opinion letters, reviews, and book chapters, were excluded.

### Information Sources and Search

Search strategies for bibliographical databases were designed by an experienced librarian (AU; Multimedia Appendix 2). We searched the Cochrane Central Register of Controlled Trials, Cochrane Database of Systematic Reviews, CINAHL with Full Text, Database of Abstracts of Reviews of Effects, Ovid MEDLINE, and Scopus in September 2018 and updated the search in December 2020. In January 2021, we also searched registries and gray literature sources: ClinicalTrials.gov, Campbell Collaboration Library, Open Science Framework, PROSPERO, BASE, Google Scholar, OpenGrey, and Science.gov. These sources were searched using variations of the terms “medical” and “photography.” The reference lists of all studies included in the full-text assessment were also searched.

### Screening

The retrieved articles were exported to and deduplicated in EndNote (Clarivate Analytics). Owing to the large number of articles for screening using a sensitive strategy that retrieved many nonrelevant articles, 2 authors (MR and TPP) screened the titles and abstracts of separate sets of articles. Articles identified in the screening were then jointly assessed by 2 authors (DŠ and MR), who discussed each article. Independent assessment by the 2 authors was not performed, and agreement indexes were not calculated as reporting in many articles was not always clear and significant disagreement was expected. The 2 assessors reached a joint conclusion on the inclusion of an article during their discussion based on the article’s full text.

### Data Charting Process

Two authors (MR and MV) created the charting form for the variables to be extracted. The form was reviewed by another author (AM) and tested by 2 authors (DŠ and MR), who extracted the data for the first 10 articles and discussed the coding for each variable. They confirmed the inclusion and exclusion criteria and then each independently extracted the data for half of the articles in the final sample. The data from 2 articles authored by some of the authors of this review were collected by the author who did not participate in the study (MV). AM checked the quality of data extraction.

### Data Items

Data were collected for the following variables: authors, article title, year of publication, source origin and country of origin, World Bank ranking of the country, publication type, journal title, journal access status, study design, study population, setting, sample size and response rate, age of the participants, gender, aim of the study, methodology, key outcomes, philosophical approach, key findings, limitations, future study ideas, and recommendations.

### Summarizing Data and Reporting Results

We summarized the data quantitatively for the description of the included studies. In the qualitative analysis, we grouped the results of the studies into main themes. According to the PRISMA-ScR guidance, we did not formally assess the methodological quality, including risk of bias, of the studies from which data were extracted as the scoping review method is not intended to be used to appraise the risk of bias of a cumulative body of evidence [20].

## Results

### Selection of Sources of Evidence

**Figure 1:**
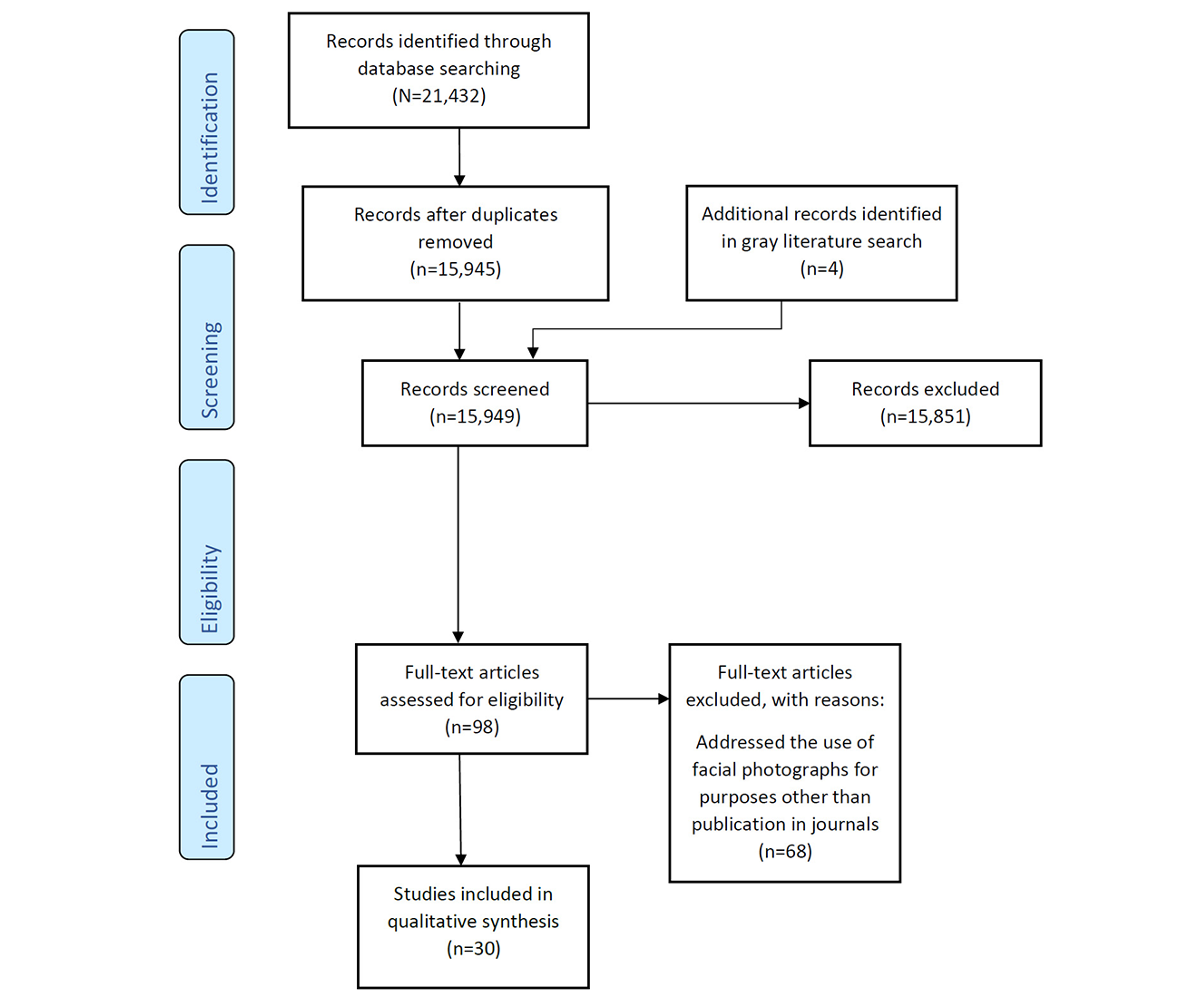
Flowchart of the literature review.

The search of bibliographical database literature retrieved a total of 21,432 published items, leaving 15,945 (74.4%) items after deduplication. The search of registries and gray literature yielded 4 additional items. After screening titles and abstracts, of the 15,949 items, 98 (0.61%) references were screened at the full-text level. We excluded 69% (68/98) of the studies as they addressed uses of facial photographs other than for publication in journals. This left 31% (30/98) of articles for analysis [2,3,6-12,14-16,21-38]. The flow diagram of the literature review is shown in Figure 1.

### Characteristics of the Sources of Evidence

Of the 30 articles included in the analysis, 6 (20%) investigated the publication of facial photographs in scientific journals as the main topic [2,3,7,32,35,36]. Other studies (24/30, 80%) had the publication of medical photography as one of the topics, so only those results were included in this review.

The included studies were published between 1994 and 2020. Almost half of the studies (13/30, 43%) were published in open access [3,6,7,11,15,21-23,25,30,31,36]. The studies were performed in countries from all continents, from both high– and low–research-intensive countries. Of the 30 studies, 29 (97%) were published in English, and 1 (3%) was published in French. Most of the studies (5/30, 17%) were conducted internationally in scientific journals, followed by studies from the United Kingdom (5/30, 17%); the United States (4/30, 13%); Australia (3/30, 10%); Brazil, Canada, France, and India (2/30, 7% each); and China, Croatia, Ireland, Nigeria, and Saudi Arabia (1/30, 3% each). The studies were published in journals from different disciplines: ethics (4/30, 13%), surgery (10/30, 33%), general medicine and education (3/30, 10%), and clinical dermatology (13/30, 43%). Most of the studies (26/30, 87%) had an observational or cross-sectional design (Table 1), 3% (1/30) were randomized controlled trials [6], 3% (1/30) used another experimental design [28], and 7% (2/30) used qualitative approaches [23,25]. Reported limitations were the small number of participants [8,12,26,27,34], pilot studies [22], a single type of health professional included [6,8,9,25,29], years of clinical practice for specialists [9], poor response rate [26,27,33,34], possibility of socially desirable answers [29,35], and a single study setting [21,22,25,29]. The limitations of the studies performed on data or journals were the small number of journals [7,36], small number of analyzed journal issues [2], filters for web-based image search, and the fluctuating number of available web-based images from academic journals [3].

There were 25 studies involving different stakeholders (Table 1): patients (n=11, 44%), legal representatives (parents) of minors (n=1, 4%), undergraduate and postgraduate students of medicine and dental medicine (n=5, 20%), nurses and other hospital health professionals (n=1, 4%), and medical doctors and doctors of dental medicine (n=11, 44%; Table 1). Another 17% (5/30) of studies involved editorial policies or published articles in journals (Table 1). The studies that involved human participants were conducted in clinical settings (17/30, 57%), at universities (3/30, 10%), and on the web (5/30, 17%). The median number of study participants was 153 (range 12-945), and the median response rate was 70% (range 17%-90%). All participants were adults, with a median age of 40 (range 27-57) years. The median percentage of women involved in the studies was 55% (range 33%-84%). The remaining 17% (5/30) of studies analyzed data on journals and articles [2,3,7,32,36].

The studies included in this review addressed three general topics: (1) ethical aspects of the use of medical photography in publications (Table 2), (2) practices and use of medical photography in journals (Table 3), and (3) characteristics of medical photographs in published articles (Table 4).

**Table 1:** Description of the studies included in the scoping review (N=30).

Study, year	Country where the study was performed	Study design	Study population	Setting or data sources	Sample size	Response rate	Age (years)
Jones [14], 1994	United Kingdom	Cross-sectional	Patients	Teaching and university hospitals or large district general hospitals (44 medical illustration departments)	35	79%	NR^a^
Cheung et al [22], 2005	United Kingdom	Cross-sectional	Patients	Emergency department	100	N/A^b^	>18
Windsor et al [38], 2006	Australia	Cross-sectional	Digital images (photographs and video clips)	Adult emergency department in Australia	493	N/A	N/A
Taylor et al [8], 2007	United Kingdom	Cross-sectional	All surgical staff	Plastic surgery units in the hospital	42	70%	NR
Clover et al [6], 2010	Ireland	Randomized controlled trial	Medical students	Medical school	126	96%	NR
Lau et al [15], 2010	United Kingdom	Cross-sectional	Patients	Department of plastic and reconstructive surgery	205	NR	>18
Engelstad et al [28], 2011	United States	Experimental study	Dental students	School of Dentistry	12	NR	Median 27 (range 24-29)
Adeyemo et al [10], 2012	Nigeria	Cross-sectional	Patients	Oral, maxillofacial, and plastic surgery clinics	338	NR	Mean 32.5 (SD 12.2; range 16-79)
Shintani and Williams [36], 2012	International	Cross-sectional	Journals	Medical journals in oral surgery	3	N/A	N/A
Devakumar et al [25], 2013	United Kingdom	Qualitative (focus groups)	Pediatricians	Teleconference via Skype	13	NR	NR
Hacard et al [29], 2013	France	Cross-sectional	Patients	Department of dermatology	272	NR	Adults: mean 57.5 (SD 17.6), children: median 1.5 (IQR 0.6-7.0), and accompanying parents: mean 35.0 (SD 6.8)
Kunde et al [31], 2013	Australia	Cross-sectional	Dermatology registrars and insurance providers	N/A	13	65%	NR
De Runz et al [24], 2014	France	Cross-sectional	Plastic surgeons and patients	Department of maxillofacial, plastic, and esthetic surgery in the hospital	176 surgeons and 93 patients	Surgeons: 42% and patients: NR	NR
Leger et al [12], 2014	United States	Cross-sectional	Patients	Hospital	398	NR	>18
Caires et al [11], 2015	Brazil	Cross-sectional	Nurses, nursing technicians, residents working at inpatient units, and physical therapists	Teaching hospital	360	Nurses: 31.4% and residents: 43.9%; regarding the place of work in the hospital, 3% worked in inpatient units	>40
Indu et al [30], 2015	India	Cross-sectional	Postgraduate students and teaching staff	Oral pathology departments	60	44%	NR
Rimoin et al [34], 2016	United States	Cross-sectional	Surgeons	Members of the American College of Mohs Surgery	158	17%	NR
Roberts et al [7], 2016	International	Cross-sectional	Journals	Medical journals that frequently publish facial photographs	13	N/A	N/A
Abbott et al [9], 2017	Australia	Cross-sectional	Dermatologists and dermatologic trainers	Australian College of Dermatologists	101	96%	NR
Dumestre and Fraulin [26], 2017	Canada	Cross-sectional	Patients, plastic surgeons, and residents	Section of plastic surgery	86 patients, 3 plastic surgeons, and 12 residents	57% of patients, 67% of surgeons, and 92% of residents	NR
Wang et al [37], 2017	China	Cross-sectional	Patients	Dermatology clinic	474	89%	Mean 31.9 (SD 11.7)
Marshall et al [3], 2018	International	Cross-sectional	Journals	Google Images and open-access articles	94	N/A	N/A
Milam and Leger [33], 2018	United States	Cross-sectional	Dermatologists	Board-certified dermatologists practicing in the United States	107	69%	Mean 47.2 (SD 11.7)
Nair et al [16], 2018	India	Cross-sectional	Patients	Ophthalmic plastic surgery clinic	280	NR	Mean 40.2 (range 18-82)
Dumestre and Fraulin [27], 2020	Canada	Cross-sectional	Plastic surgeons, residents, and patients	Section of plastic surgery	16 plastic surgeons, 24 residents, and 84 patients and parents	51% of surgeons and residents and 56% of patients	NR
Lessing et al [32], 2019	International	Cross-sectional	Journals	Top 10 impact factor general medical journals	10	N/A	N/A
Abouzeid et al [21], 2020	Saudi Arabia	Cross-sectional	Dental students	School of Dentistry	233	86%	NR
Costa et al [23], 2020	Brazil	Qualitative study (semistructured interviews)	Dentists	Unclear (clinical setting)	52	NR	Mean 30.4
Roguljić et al [35], 2020	Croatia	Cross-sectional	Patients, students of medicine and dentistry, and doctors of medicine and dental medicine	Dental outpatient clinics	292 patients, 281 students, and 281 doctors	Patients: NR, physicians: 85%, medical students: 72%, and dental students: 58%	Patients: median 55 (IQR 22), students: median 23 (IQR 1), and physicians: median 40 (IQR 18)
Roguljić et al [2], 2022	International	Cross-sectional	Journals	Medical journals in dentistry and otolaryngology	103	N/A	N/A

^a^NR: not reported.

^b^N/A: not applicable.

**Table 2:** Ethical aspects of medical photography for publications.

Study, year	Study aim	Key findings
Jones [14], 1994	To determine common practices and attitudes toward medical photography among hospital patients	Most departments insist on written informed consent. When releasing clinical slides for publication, most departments insist that patient consent is obtained.
Cheung et al [22], 2005	To investigate patients’ attitudes toward medical photography and consent use at an emergency department	Most participants gave consent for publication of images in a medical journal or books but were more likely to refuse consent for use of images on internet medical sites.
Taylor et al [8], 2007	To investigate awareness of and compliance with present regulations regarding clinicians taking digital photographs of patients	Less than half of the surgeons reported always obtaining consent, more often verbal than written for different purposes. The process of consent must include the option that consent may be withdrawn at any time before the information has passed irretrievably into the public domain.
Lau et al [15], 2010	To explore patient perception of digital photography	Approximately half of the patients would consent for each purpose of use.
Adeyemo et al [10], 2012	To determine acceptance and perception of medical photography among Nigerian patients	Most respondents indicated that their consent should be sought for each purpose.
Kunde et al [31], 2012	To review ethical and legal considerations of clinical photography in dermatology and present a hypothetical medicolegal scenario	Verbal consent would be commonly obtained for different purposes, including publication.
Devakumar et al [25], 2013	To explore the issues around photography in low-resource settings by conducting discussions with medical doctors and researchers who are currently working or have recently worked in low-resource settings with children	Participants considered that informed consent is required, but its form may vary depending on the context. Protection of the rights of children is especially important in relation to photographs.
Hacard et al [29], 2013	To evaluate patients’ perceptions of medical photographs	Written consent was considered necessary for adult and pediatric patients.
De Runz et al [24], 2014	To analyze the use of photography by plastic surgeons, the perception of this use by the patients, and medicolegal and ethical consequences	Most of the surgeons considered that verbal consent or no consent is sufficient for taking patient photographs.
Leger et al [12], 2014	To investigate patient opinions on clinical photography	Respondents preferred permission for photographs to be obtained in written form.
Caires et al [11], 2015	To evaluate the knowledge of health care professionals regarding taking medical photographs within the hospital environment among hospital staff	Verbal and written consent for taking the photographs was lacking.
Indu et al [30], 2015	To assess the awareness of oral pathologists regarding various aspects of medical photographs	Most students and faculty members informed the patients of the purpose of the photograph and took verbal consent. Most of them mentioned to the patient their right to withdraw consent.
Rimoin et al [34], 2016	To elucidate the nature of use, storage, and informed consent for digital photography among Mohs surgeons	A very small number of responders pursued some form of consent before taking photographs, with most preferring verbal consent over written consent. They considered that consent should be obtained for different purposes.
Abbott et al [9], 2017	To evaluate the understanding of the use of smartphones in clinical practice regarding professional and legal risks	Patient consent was not often documented regarding different uses of patient photographs; respondents mostly did not receive information on relevant guidelines.
Dumestre and Fraulin [26], 2017	To evaluate a smartphone app for clinical photography regarding patient security among plastic surgeons, plastic surgery residents, and patients who had undergone plastic surgery	The app ensured adequate consent for educational and research purposes but was inadequate for publication and disclosure to the public.
Wang et al [37], 2017	To assess the perception and acceptability of medical photography in patients of dermatology	Almost half of the respondents considered that oral consent only should be obtained before taking medical photographs, whereas the other half of the respondents answered that written consent should be obtained. Most of the respondents argued that all possible image uses should be detailed in the consent form.
Milam and Leger [33], 2018	To examine dermatologists’ current practices in medical photography	Most respondents agreed that patients should be allowed to withdraw consent and should be informed of the use of their photographs on each occasion, including publication.
Dumestre and Fraulin [27], 2020	To evaluate a smartphone app for clinical photography that prioritizes and facilitates patient security	Patients considered the consent process acceptable in the app. Surgeons and residents felt that the consent process was superior or equivalent to previous methods.
Costa et al [23], 2020	To evaluate the behavior of dentists on the use of patients’ images	Participants considered that informed consent for sharing patients’ images, including in publications, can be verbal or absent when the patient cannot be identified.
Roguljić et al [35], 2020	To explore opinions of patients, students, and doctors on the acceptability of different levels of deidentification and the informed consent needed for publication in academic journals	All respondents reported increased preference for more stringent forms of permission as the level of identifiability in photographs increased.

**Table 3:** Practices and use of medical photography for research publications.

Study, year	Study aim	Key findings
Jones [14], 1994	To determine common practices and attitudes toward medical photography among hospital patients	Most respondents felt that, even though the patient was consenting to treatment by being in hospital, they still had a right to refuse to be photographed.
Windsor et al [38], 2006	To summarize 3 months of digital photography taking in an adult emergency department	The use of digital photographs and video clips in clinical settings is very useful in creating a database of confidential medical records that can be used for medical teaching and publication.
Taylor et al [8], 2007	To investigate awareness of and compliance with present regulations regarding clinicians taking digital photographs of patients	Patients considered themselves insufficiently informed of their right to withdraw consent. Surgeons used methods of deidentification for patient photographs for teaching and publication purposes. They stored password-protected photographs in PCs and personal cameras.
Clover et al [6], 2010	To analyze the effectiveness of blacking out the eyes in facial photographs through alternative techniques	Deidentification failed most in the group with a covered eye area in a photograph, followed by covering the eye and nose and covering the eyes, nose, and mouth.
Lau et al [15], 2010	To explore patients’ perception of digital photography	Patients preferred the use of hospital cameras and nonidentifiable photographs for all purposes.
Engelstad et al [28], 2011	To test the hypothesis that unaltered features from an original full-face patient image could be blended with other facial images to create a unique facial composite that deidentifies the patient	Facial composites were more effective at deidentification than traditional methods.
Adeyemo et al [10], 2012	To determine acceptance and perception of medical photography among Nigerian patients	Patients had high acceptance of medical photography, especially of nonidentifiable photographs. The use of institutional cameras operated and stored by the patients’ physicians was the preferred method.
Kunde et al [31], 2012	To explore ethical and legal considerations of clinical photography in dermatology and present a hypothetical medicolegal scenario	Dermatologic registrars used personal smartphones for taking photographs for different purposes, such as to obtain advice from peers, teaching, sharing with colleagues, treatment and disease monitoring, and publication.
Devakumar et al [25], 2013	To explore the issues around photography in low-resource settings	Photographs of children in medical and research settings are useful as they enrich teaching, research, and advocacy.
Hacard et al [29], 2013	To evaluate patients’ perceptions of medical photography	Low acceptability of the use of the images in professional emails, health magazines, television health programs, and medical websites. Publication in medical scientific articles was significantly more acceptable for adults than for children.
De Runz et al [24], 2014	To analyze the use of medical photography by plastic surgeons and perception of this use by the patients	Patients and surgeons had high acceptance of taking medical photographs for diagnosis and treatment follow-up and lower acceptance for publication purposes.
Leger et al [12], 2014	To investigate patients’ opinions of clinical photography	Nonidentifiable photographs taken by their physician with clinic-owned cameras within the institution for all purposes were preferred. Race and ethnicity, income level, and age influenced the patients’ answers.
Abbott et al [9], 2017	To evaluate the understanding of the use of smartphones in clinical practice regarding professional and legal risks	Most respondents had and used smartphones for taking medical photographs for different purposes.
Dumestre and Fraulin [26], 2017	To evaluate a smartphone app for clinical photography regarding patient security among plastic surgeons, plastic surgery residents, and patients who had undergone plastic surgery	Patients: high acceptance of use for educational, research, communication, and medical documentation purposes and less acceptance for publication in a public medium; surgeons and residents: the app will be suitable for use when certain issues regarding consent and protection of confidentiality are overcome
Wang et al [37], 2017	To assess the perception and acceptability of medical photography in patients of dermatology	Patients’ physicians using clinic-owned cameras were the most accepted as photographers. Low acceptability of use was reported for medical websites and televised health programs.
Milam and Leger [33], 2018	To examine the current medical photography practices of dermatologists	Respondents reported the use of medical photographs for different purposes, including research and publication. They used digital cameras, personal phones, and electronic medical record applications. Photographs were stored in the office computer with various security measures and shared via email with colleagues and patients.
Nair et al [16], 2018	To assess patient perceptions regarding medical photography and smart devices	Most patients accepted the use of smartphones for medical photography, but only a third approved the use of medical photographs in presentations and medical journals. Patients preferred to be photographed by their physician with their own camera or an institutional camera at the institution.
Dumestre and Fraulin [27], 2020	To evaluate a smartphone app for clinical photography that prioritizes and facilitates patient security	Patients: the purpose of the app was well explained, and it was perceived as safe; surgeons and residents: respondents believed the app was suitable for broad implementation to receive and send patient photographs
Abouzeid et al [21], 2020	To evaluate the awareness of practice, opportunity, and morals of dental photography among undergraduate dental students	Almost all students take photographs on a regular basis. Phone cameras were the most commonly used device, followed by digital single-lens reflex cameras. Verbal consent was obtained before taking photographs. For research publication, they edited the photographs using specific software or by covering the eye area. More training in photography techniques was perceived to be necessary.
Costa et al [23], 2020	To evaluate the behavior of dentists in using patients’ images	The most common purposes of the use of photographs were didactic or academic. Discussion groups on social media may increase the knowledge of the use of patient photographs.

**Table 4:** Medical photography in research publications.

Study, year	Study aim	Key findings
Shintani and Williams [36], 2012	To investigate how guidelines on the protection of patient anonymity are actually implemented and how effective such methods of protection are in 3 oral surgery journals	Most of the published photographs were of the entire face or a part of the face. Masking the eye area was observed in half of the facial photographs, and deidentification failed.
Marshall et al [3], 2018	To analyze current practices used in patient facial photograph deidentification	Sensitive medical photographs from articles freely available were found on Google Images. A small number of articles reported obtaining written informed consent for publication of medical images from patients undergoing transgender surgery.
Roberts et al [7], 2016	To analyze the current practices used in patient facial photograph deidentification and set forth standardized guidelines for improving patient autonomy that are congruent with medical ethics and health insurance	Facial image anonymization guidelines varied across journals. When anonymization was attempted, 87% of the images were inadequately concealed. The most common technique used was masking the eyes alone with a black box.
Lessing et al [32], 2019	To assess consent requirements in a sample of 10 top impact factor general medicine journals that publish clinical images	All journals had web-based information regarding clinical image consent requirements. Written consent was required for all identifiable photographs. No journals were fully compliant with ICMJE^a^ consent recommendations.
Roguljić et al [2], 2022	To analyze policies of journals that publish research and their implementation regarding patient consent for facial image publication	Only approximately half of the analyzed journals had a specific policy on clinical images. A small number of articles that published recognizable patient facial images included a statement on consent for image publication.

^a^ICMJE: International Committee of Medical Journal Editors.

### Ethical Aspects of Medical Photography for Research Publications

Almost all studies that analyzed the ethical aspects of medical photography in research publications (19/20, 95%; Table 2) reported that the consent process was not conducted properly for different uses of patients’ photographs, including for journal publications. A total of 5% (1/20) of the studies stated that they addressed medicolegal issues [31], but the study findings were not put in the context of privacy protection legal regulations. Studies that included patients and health professionals (13/20, 65%) were affirmative of the practice of obtaining informed consent for the use of patient medical photographs. However, relevant stakeholders recognized different levels of potential risks if patient medical photographs were used for different purposes, from treatment planning and follow-up in medical documentation to education and different forms of publication.

Studies that investigated patients’ perspectives on the importance of informed consent (8/20, 40%) showed that patients were aware of the increased risks of being recognized after the publication of their medical photographs by anyone who has access to the publication [10,12,14-16,22,35,37]. Patients in an emergency medicine department were more likely to refuse consent for the use of images on internet sites, but they would provide consent for the purposes of medical education, medical books, or journals [22]. Patients in that study were not aware that medical books or journals could also be accessed on internet sites [22]. Patients in a plastic surgery department were more likely to approve the use of medical photography for diagnosis and treatment follow-up but were also less likely to consent for publication purposes [24]. A total of 10% (2/20) of the studies showed that patients preferred to be offered consent for a specific purpose and not a general consent for any type of use of their medical photographs, including identifiable and nonidentifiable photographs [10,14]. Furthermore, patients preferred being offered a written consent form rather than being offered oral consent [12,14,29].

Studies that involved plastic surgeons and dentists showed diversity in written informed consent for taking photographs of patients. A total of 17% (5/30) of the studies showed that patient consent was not always obtained for taking and using patient medical photographs and that the prevalent opinion was that verbal consent was sufficient [3,8,23,24,26]. Studies that involved dermatologists (2/20, 10%) [9,33] showed that most of them did obtain consent for patients’ photographs, but they did not consider it necessary. In addition, dermatologists emphasized the need for better education on this issue and the need to create more realistic and practical policies for everyday practice. They also asked for better policies and tools for patients to exercise their right to withdraw their consent at a later time.

A small number of studies (4/20, 20%) investigated other health professionals’ opinions regarding ethical publishing of medical photography, involving residents, students, nurses, nursing technicians, and physical therapists [11,30,31,35]. These studies reported that health professionals in general had a lack of knowledge regarding the need to obtain patient written consent and the use of patient medical photographs in general. A survey of nursing staff, physical therapists, and physicians reported a lack of knowledge of both verbal and written consent for taking patient photographs [11], whereas undergraduate and postgraduate students considered that verbal consent was sufficient for medical image publication [30,35]. Similarly, in the study by Kunde et al [31], 4 out of 13 dermatology registrars reported that they used verbal consent for taking photographs of patients for publishing purposes.

In total, 7% (2/30) of the studies investigated the issues of taking and using medical photographs of children [25,29]. In the focus group study by Devakumar et al [25], pediatricians emphasized that, although photographs are valuable resources, they might be potentially harmful. Thus, written informed consent was considered mandatory. In addition, they thought that the publication of photographs from this patient population required more stringent forms of informed consent to protect children. Similarly, a questionnaire survey by Hacard et al [29] included patients from a dermatology department and parents or legal guardians from the pediatric department and showed that acceptance of medical photographs was high among both groups. They considered that written informed consent was required for each purpose of use, with participants from the pediatric department being stricter in this aspect.

### Practices and Use of Medical Photography for Research Publications

Studies addressing practices for taking medical photographs (20/30, 67%; Table 3) were conducted among different stakeholders: patients, medical staff, graduate and postgraduate students of medicine and dental medicine, residents, dermatologists, dentists, and plastic surgeons. The devices used for taking medical photographs included institutional cameras, personal cameras, and smartphones [9,29,38]. The device most often used for taking patient photographs was a personal camera (smartphone) [8,9,16,21,29,33], but 10% (3/30) of the studies showed that patients preferred to be photographed by their physicians using institutional cameras and in an institutional setting [10,15,37]. Patients considered that the use of personal devices, particularly smartphones, for taking medical photographs constituted a potential breach of patient-physician confidentiality [16].

Patients and physicians showed a high level of acceptance of medical photography for different purposes, such as medical documentation, research, communication, and education, but less for their publication in a public medium such as medical websites, professional emails, health magazines, and television health programs [24,26,29,37]. Furthermore, they preferred nonidentifiable over identifiable photographs for all types of use [10,12,15,35]. However, the studies also showed that conventional methods of deidentification of facial photographs concealing the eye area are not sufficient to achieve nonidentifiability [6,7]. The exception was the study by Engelstad et al [28], which demonstrated successful deidentification using a blended facial composite technique. This technique combined significant components of the original patient’s photograph with cropped parts of the head and neck from other photographs using a computer software program to create nonidentifiable photographs that still presented patient details relevant to the clinical findings.

Several studies (8/20, 40%) reported that both patients and physicians considered important to ensure secure data storage, maintenance of privacy, and controlled access to the images [8,9,14,16,26,29,30,37]. A total of 7% (2/30) of the studies reported that dental students and dentists lacked knowledge and training regarding the techniques of taking and managing patient photographs, including for the purpose of publishing [21,23].

### Medical Photography in Journal Articles

Studies addressing practices of publishing medical photographs in medical journals (5/30, 17%; Table 4) demonstrated a large diversity in publishing practices regarding consent for publication. Studies that analyzed high-impact general medical journals [32] or journals publishing dentistry and otorhinolaryngology research [2] showed that journal policies regarding the consent process and consent forms were insufficient and that existing ethical professional guidelines were not fully implemented in actual practices. A total of 40% (2/5) of the studies analyzed the deidentification of facial photographs published in medical journals and showed that the most common techniques, such as concealing the eye area, were not sufficient to protect the patient’s identity [7,36]. The authors of these studies emphasized the importance of improving the policies regarding consent for publication of patient facial photographs. All facial photographs, with or without the applied deidentification technique, should require separate written consent for publication from the patient.

The only study that analyzed web access to patient-sensitive data published in open-access journals and platforms was the study by Marshall et al [3]. This study showed that patient medical photographs, including the face (8.1% of the photographs), were published in open-access formats and could be accessed easily via a Google Image browser, indicating a serious lack of protection of patient-sensitive data.

## Discussion

### Principal Findings

Our scoping review identified 30 studies that investigated different aspects of publishing identifiable patient photographs in research journals over a period of >25 years. It seems that, despite existing legal and professional guidelines regarding the use of patient photographs, obtaining informed consent properly is a challenge for many health professionals not only for scientific publications but also for other purposes. Relevant stakeholders did not always consider that only written informed consent was necessary for the publication of a patient photograph, and they also considered that it was possible to use patient photographs after obtaining oral consent or even without consent. Although relevant stakeholders were aware of the potential issues of using patients’ medical photographs in terms of violating privacy and confidentiality, they did not have a satisfactory level of knowledge, skills, or tools to put existing guidance on medical photography in research into practice. Finally, there was little awareness of the current challenges, such as the protection of patients’ clinical images and their permanence and availability in a digital environment.

Our study had some limitations. Most of the studies included in this scoping review (22/30, 73%) did not investigate medical photography publishing as the primary topic but as one of several purposes and aspects of medical photography. In addition, the studies did not define clear criteria for the deidentification of facial photographs. Although our search strategy did not have language restrictions, 97% (29/30) of the included studies were in English, and it is possible that there are studies in other languages that were not captured by the search of standard databases, registries, and gray literature sources. The methodological issues of the studies, such as questionnaire survey designs and insufficient reporting of methods and results, which might influence the validity of the studies, also limit the comparisons and generalizability of the findings.

We also did not assess the compliance of practices with legal standards as they varied in the countries in which the studies were performed. We presumed that the studies considered the contemporary legal regulations and investigated the compliance, practices, and opinions of the participants in relation to these regulations. The protection of patients’ personal data is ensured by strict legal regulations such as the Health Insurance Portability and Accountability Act in the United States [39]. In Europe, the General Data Protection Regulation (GDPR) provides the strictest protection of patient personal data, including for research purposes [40,41]. According to the GDPR, consent is one of the legal bases for lawful processing of personal data and is most relevant for publications in scientific journals. Accordingly, a research participant consents to have the right to be informed, withdraw consent, have access to data, rectify the data or erase them, restrict data processing, and obtain and reuse their data (data portability) [40,41].

The problem of following procedures for obtaining valid patient consent in clinical practice is not limited to the purposes of publication but is a part of the general challenge in medical practice. A recent updated systematic review by Glaser et al [42] regarding interventions to improve patient comprehension in obtaining informed consent for medical procedures showed that, although progress was achieved, the consent process does not always meet 4 key elements of valid informed consent—decision capacity, documentation of consent, disclosure, and competency. The findings of our scoping review focused on the ethical publication of patient photographs in scientific journals and also showed that there are problems not only with the existing guidance, such as successful deidentification of published photographs, but even more with implementing existing guidance in practice, particularly in relation to proper and adequate informed consent. For example, despite the existence of ethical guidelines created by relevant professional or governmental organizations on the importance of obtaining patient consent in written form [5,43], studies that investigated the practices of obtaining informed consent for different uses of medical photographs (7/30, 23%) showed that even high-profile academic clinicians still considered verbal consent sufficient [8,9,23,26,33,35,37]. In another study, dermatologists reported that they did not always document patient consent for a specific purpose [9], indicating the need for better education at all career stages as well as the creation of more practical guidance for the implementation of standards for consent procedures in everyday practice. An unsatisfactory level of knowledge was also present among other health professionals as well as among patients [9,11,24,30,35]. Inasmuch as most of the analyzed studies in our scoping review used cross-sectional designs (26/30, 87%), future studies should have an interventional or qualitative design to investigate possible solutions for increasing the level of knowledge of all relevant stakeholders. We also did not identify studies that investigated whether consent for a research study included consent for publication and whether that consent provided sufficient information to the participants regarding how their photographs would be published and under which publishing license.

As open-access publishing has become a common format for medical research, patients’ informed consent for identifiable photographs in open-access journals deserves special attention. Considering that patients can be a very heterogeneous group regarding educational level and socioeconomic background, health professionals should be able to explain to them that scientific articles published in open-access journals could be as accessible as any other information on the internet. It seems that patients do not always perceive that scientific journals are available on the web in the same way as any other content on the internet. For example, patients in an emergency department were more likely to consent to the use of their medical photographs in medical publications than on websites [22]. Thus, it is important that patients are fully informed of the implications of publishing photographs in a web-based medium before signing the consent form. They should be warned that open-access formats allow access to their photographs without any safeguards, leaving no possibility to withdraw or control their future use.

Generally, both patients and health professionals had high acceptance of medical photography and found it useful for many purposes, but patients preferred the use of nonidentifiable photographs [10,12,15]. Several studies that analyzed methods of deidentification of facial photographs (4/30, 13%) showed that conventional techniques were insufficient, and such photographs cannot be considered as nonidentifiable [6,7,35,36]. Such photographs should be considered as potentially reidentifiable, which leads to the conclusion that, in many situations, it is not possible to determine whether the photograph is identifiable. Furthermore, different computer programs have been developed to identify a person from a photograph (eg, DeepFace, Visual Search, Social Mapper, and Amazon Rekognition) with high levels of accuracy [44]. Patients may not be aware of this issue, but physicians should anticipate such situations and protect patients by providing proper informed consent. Although health care professionals commonly use medical photography in medical documentation, patient privacy becomes jeopardized when such photographs are used for other purposes such as communication with colleagues, lectures, presentations, or publications [43]. Patients were more likely to allow the use of their photographs for medical documentation, treatment follow-up, and education than for publications, websites, social media, and televised programs [26,29,37]. These findings suggest that patients recognize the increased risk of violating their privacy in a public environment regardless of their general affirmative attitudes toward medical photographs.

The studies included in this review addressed not only fully identifiable photographs but also potentially reidentifiable photographs as well as those that were considered to be nonidentifiable. The distinction between these types of photographs is very difficult [6-8], and it has been shown that patients and their families or social environment can recognize them even if the photograph that was published was considered to be fully nonidentifiable [45]. It would be safe to consider that all photographs of a patient’s body are potentially identifiable or reidentifiable and that consent for the publication of such photographs should be sought.

The full maintenance of medical photography integrity requires practical protocols that should be in accordance with current guidelines and best ethical practices [5,46]. However, there are still different practices for taking and storing photographs, so it seems that the process of taking patient photographs has not yet been standardized and might be one of the reasons why the consent processes for their different uses are not often performed and reported in line with best ethical practices. Studies that analyzed the clinical practices of taking medical photographs (20/30, 67%) showed that patients were more likely to be photographed by their physicians than by other health personnel. In addition, patients were more consenting of being photographed with institutional cameras than with personal devices. These findings indicate that taking an identifying photograph is a sensitive procedure in which patients expect a high level of confidentiality and privacy. Following established guidelines such as those from the ICMJE would be a good beginning toward the responsible publishing of medical photography.

The analysis of medical journals also demonstrated the problem of an insufficient consent process for the publication of patient photographs. As journals have been shown not to be fully compliant with ICMJE consent recommendations, it was recommended that standard consent forms for the publication of identifiable images in medical journals should be developed [32]. Studies that investigated the ethical publication of identifiable photographs of patients (5/30, 17%) came to a very similar conclusion: there is a lack of consensus from journal editors and publishers, and uniform publishing policies are needed. The aforementioned recommendation seems reasonable and actionable as editorial organizations have created similar standards for other declarations in published studies, such as competing interest declarations from the ICMJE [47]. As previously mentioned, the digital environment represents a new challenge for publishing practices, especially with the growing trend of open-access publications. Journal editors and publishers should make clear what their publishing practices involve with regard to the use and sharing of published patient photographs, develop appropriate procedures for adequate and responsible declaration of obtaining informed consent for photograph publication that are separate from declaring and describing informed consent for research, and incorporate the submission of relevant declarations in web-based manuscript submission systems. They also have to protect the identity of the patients and not receive or publish consent forms from patients but rather ensure that authors provide declarations that appropriate procedures were followed and that patients gave informed consent for publishing their (identifying) photographs. If journals advise authors to provide proof of consent for persons mentioned in the acknowledgments [5], then they have to ensure the integrity of publishing patient photographs.

### Gaps in Knowledge

This scoping review provided information about the attitudes, opinions, and practices regarding medical photography among relevant stakeholders and showed that they recognized the issues of privacy protection when medical photographs are used, particularly in publications. The impact of recent legal regulations related to personal data protection, such as the GDPR [40,41], on the publication of potentially identifiable photographs of research participants needs also to be further investigated. As it was shown that all stakeholders lack knowledge regarding the ethical publication of patient medical photographs, interventional studies are needed to address effective education and training. In addition, there is no evidence in the literature of the knowledge of stakeholders regarding published medical photographs in freely available web-based formats, particularly those published under licenses for wide use. An emerging issue that has not yet been addressed is the publication of medical photographs in preprints. Preprints, as “complete and public drafts of scientific documents, not yet certified by peer review” [48], do not pass the same scrutiny as regular journal publications, but their number and importance have enormously increased [49,50]. The latest update of the ICMJE Recommendations for the Conduct, Reporting, Editing, and Publication of Scholarly Work in Medical Journals [5] in 2021 emphasized the need for appropriate declarations regarding published articles in preprint archives, such as disclosure of funding sources and disclosure of interest, but ethical issues about consent for patients’ photographs were not mentioned. As one of the main aims of preprints is to increase the discoverability of research, the openness of such publications may be a facilitator for the research community but a threat and concern for patients whose photographs may be published in a way that will hinder the protection of their privacy. A recent study of editorial policies in preprint archives did not report on patient privacy protection, and only 20% of the archives in health sciences stated that they followed the ICMJE recommendations [51]. Future studies should investigate the practices of publishing patient photographs in preprints.

### Conclusions

This scoping review of opinions, standards, and practices in publishing identifiable patient photographs in almost a 30-year period leads to the conclusion that all stakeholders in this issue have not fully developed and implemented best-practice standards for publishing medical images, particularly identifiable photographs of individuals. They are also not ready for the challenges of new developments in how we communicate research. In a digital environment, the protection of patient privacy is especially difficult because of how research information is shared on the web and on social media. Furthermore, newly developed digital tools for the deidentification of photographs are not commonly used, although it is clear that a standard black tape across the eyes on a photo does not make the person nonidentifiable. Despite the existence of legal, governmental, and professional policies and guidelines, the consent process and obtaining informed consent for publication are often not properly conducted or adequately reported in scientific literature. Relevant professional and ethics organizations, as well as journals and publishers, should address the emerging challenges in privacy protection by developing and updating guidance, protocols, and tools to ensure best practices in publishing patient photographs in medical literature.

## Appendix

Multimedia Appendix 1 PRISMA-ScR (Preferred Reporting Items for Systematic Reviews and Meta-Analyses extension for Scoping Reviews) checklist.

Multimedia Appendix 2 Search strategies.

## Abbreviations

GDPR: General Data Protection Regulation
ICMJE: International Committee of Medical Journal Editors
PRISMA-ScR: Preferred Reporting Items for Systematic Reviews and Meta-Analyses extension for Scoping Reviews

## Data Availability

The data sets used and analyzed during this study are available from the corresponding author upon reasonable request.
