# Supplementary material for: Publishing Identifiable Patient Photographs in Scientific Journals: Scoping Review of Policies and Practices
Source: J Med Internet Res. 2022 Aug 31;24(8):e37594. doi: 10.2196/37594 (PMC9475410; doi:10.2196/37594)
Supplement: Multimedia Appendix 2 [file jmir_v24i8e37594_app2.pdf]

Roguljić et al. Publishing identifiable patient photographs in scientific journals: a scoping review of policies and practices
Supplementary information: Search strategies

Database: EBM Reviews - Cochrane Database of Systematic Reviews <2005 to September 5, 2018>
Search Strategy:
----
1 photography.kw. (2)
2 ("diagnostic techniques and procedures" or diagnostic imaging or cardiac imaging techniques or neuroimaging or radiography or tomography or ultrasonography or whole body imaging).kw. (109)
3 photograph$.tw. (189)
4 imag$.tw. (1981)
5 ((medical or clinical or patient or dental) adj3 (record$ or data)).tw. (3842)
6 ((eye$ or face or facial) adj3 mask$).tw. (119)
7 ((eye$ or face or facial) adj3 blurr$).tw. (24)
8 black stripe.tw. (0)
9 or/1-8 (5110)
10 (confidentiality or informed consent).kw. (5)
11 privacy.kw. (0)
12 ((patient or written or verbal or informed) adj3 consent).tw. (429)
13 (patient adj3 (privacy or confidentiality or agreement or anonymity)).tw. (37)
14 (bioethics or ethics, clinical or ethics, research or professional misconduct).kw. (0)
15 ((bioethic$ or ethic$) adj3 (standard$ or implicat$ or responsib$ or dilemma$ or problem$)).tw. (100)
16 or/10-15 (528)
17 publishing.kw. (7)
18 (publications or teaching materials).kw. (5)
19 publish$.tw. (10789)
20 (publication$ or journal$ or textbook$ or handbook$ or presentation$ or poster$).tw. (10499)
21 or/17-20 (10800)
22 9 and 16 and 21 (331)

****

Database: EBM Reviews - Cochrane Central Register of Controlled Trials <August 2018>
Search Strategy:
----
1 exp Photography/ (8376)
2 "diagnostic techniques and procedures"/ or diagnostic imaging/ or exp cardiac imaging techniques/ or exp neuroimaging/ or exp radiography/ or exp tomography/ or exp ultrasonography/ or whole body imaging/ (40494)
3 photography.kw. (765)
4 photograph$.tw. (4534)
5 diagnostic techniques.kw. (13)
6 (imaging or neuroimaging or radiography or tomography or ultrasonography).kw. (19655)
7 imag$.tw. (39700)
8 ((medical or clinical or patient or dental) adj3 (record$ or data)).tw. (28481)
9 ((eye$ or face or facial) adj3 mask$).tw. (1352)
10 ((eye$ or face or facial) adj3 blurr$).tw. (56)
11 black stripe.tw. (0)
12 or/1-11 (110274)
13 confidentiality/ or exp informed consent/ (691)
14 exp Privacy/ (75)
15 (confidentiality or informed consent or privacy).kw. (4040)
16 ((patient or written or verbal or informed) adj3 consent).tw. (13616)
17 (patient adj3 (privacy or confidentiality or agreement or anonymity)).tw. (264)
18 exp bioethics/ or exp ethics, clinical/ or ethics, research/ or exp professional misconduct/ (220)
19 (bioethics or ethics, clinical or research ethics or professional misconduct).kw. (406)
20 ((bioethic$ or ethic$) adj3 (standard$ or implicat$ or responsib$ or dilemma$ or problem$)).tw. (279)
21 or/13-20 (15558)
22 exp Publishing/ (143)
23 exp publications/ or exp teaching materials/ (4586)
24 (publishing or publication$ or teaching materials).kw. (1875)
25 publish$.tw. (25635)
26 (publication$ or journal$ or textbook$ or handbook$ or presentation$ or poster$).tw. (39380)
27 or/22-26 (66016)
28 12 and 21 and 27 (372)
****

CINAHL with Full Text (EBSCOhost)

S22 S9 AND S16 AND S21 288
S21 S17 OR S18 OR S19 OR S20 401,981
S20 TI (publication* or journal* or textbook* or handbook* or presentation* or poster*) OR AB (publication* or journal* or textbook* or handbook* or presentation* or poster*) 126,632
S19 TI publish* OR AB publish* 88,821
S18 MH "Publications+" OR MH "Teaching materials+" 84,898
S17 MH "Publishing+" 157,432
S16 S10 OR S11 OR S12 OR S13 OR S14 OR S15 24,658
S15 TI ((bioethic* or ethic*) N3 (standard* or implicat* or responsib* or dilemma* or problem*)) OR AB ((bioethic* or ethic*) N3 (standard* or implicat* or responsib* or dilemma* or problem*)) 6,128
S14 MH "bioethics" OR MH "ethics, clinical+" OR MH "ethics, research" OR MH "professional misconduct+" 9,496
S13 TI (patient N3 (privacy or confidentiality or agreement or anonymity)) OR AB (patient N3 (privacy or confidentiality or agreement or anonymity)) 2,183
S12 TI ((patient or written or verbal or informed) N3 consent) OR AB ((patient or written or verbal or informed) N3 consent)   7,608
S11 MH "Privacy+" 1
S10 MH "confidentiality" OR MH "informed consent+" 6
S9 S1 OR S2 OR S3 OR S4 OR S5 OR S6 OR S7 OR S8 277,969
S8 TI (black stripe) OR AB (black stripe) 3
S7 TI ((eye* or face or facial) N3 blurr*) OR AB ((eye* or face or facial) N3 blurr*) 61
S6 TI ((eye+ or face or facial) N3 mask*) OR AB ((eye+ or face or facial) N3 mask*) 638
S5 TI ((medical or clinical or patient or dental) N3 (record* or data)) OR AB ((medical or clinical or patient or dental) N3 (record* or data)) 70,360
S4 TI (imag*) OR AB (imag*) 87,526
S3 TI (photograph*) OR AB (photograph*) 5,007
S2 MH ("diagnostic techniques and procedures") OR MH "diagnostic imaging" OR MH "cardiac imaging techniques+" OR MH "neuroimaging+" OR MH "radiography+" OR MH "tomography+" OR MH "ultrasonography+" OR MH "whole body imaging" 163,715
S1 MH "Photography+" 56,100

Database: EBM Reviews - Database of Abstracts of Reviews of Effects <1st Quarter 2016>
Search Strategy:
----
1 photography.kw. (11)
2 ("diagnostic techniques and procedures" or diagnostic imaging or cardiac imaging techniques or neuroimaging or radiography or tomography or ultrasonography or whole body imaging).kw. (1461)
3 photograph$.tw. (28)
4 imag$.tw. (1409)
5 ((medical or clinical or patient or dental) adj3 (record$ or data)).tw. (1607)
6 ((eye$ or face or facial) adj3 mask$).tw. (20)
7 ((eye$ or face or facial) adj3 blurr$).tw. (1)
8 black stripe.tw. (0)
9 or/1-8 (3715)
10 (confidentiality or informed consent).kw. (16)
11 privacy.kw. (1)
12 ((patient or written or verbal or informed) adj3 consent).tw. (41)
13 (patient adj3 (privacy or confidentiality or agreement or anonymity)).tw. (6)
14 (bioethics or ethics, clinical or ethics, research or professional misconduct).kw. (2)
15 ((bioethic$ or ethic$) adj3 (standard$ or implicat$ or responsib$ or dilemma$ or problem$)).tw. (12)
16 or/10-15 (61)
17 publishing.kw. (7)
18 (publications or teaching materials).kw. (19)
19 publish$.tw. (9321)
20 (publication$ or journal$ or textbook$ or handbook$ or presentation$ or poster$).tw. (12034)
21 or/17-20 (14271)
22 9 and 16 and 21 (14)

****

Database: Ovid MEDLINE(R) and Epub Ahead of Print, In-Process & Other Non-Indexed Citations and Daily <1946 to September 06, 2018>

Search Strategy:

----

1 exp Photography/ (535245)
2 "diagnostic techniques and procedures"/ or diagnostic imaging/ or exp cardiac imaging techniques/ or exp neuroimaging/ or exp radiography/ or exp tomography/ or exp ultrasonography/ or whole body imaging/ (1812852)
3 photograph$.tw. (47965)
4 imag$.tw. (1028967)
5 ((medical or clinical or patient or dental) adj3 (record$ or data)).tw. (296191)
6 ((eye$ or face or facial) adj3 mask$).tw. (4020)
7 ((eye$ or face or facial) adj3 blurr$).tw. (327)
8 black stripe.tw. (37)
9 or/1-8 (2666354)
10 confidentiality/ or exp informed consent/ (58010)
11 exp Privacy/ (14071)
12 ((patient or written or verbal or informed) adj3 consent).tw. (36267)
13 (patient adj3 (privacy or confidentiality or agreement or anonymity)).tw. (3142)
14 exp bioethics/ or exp ethics, clinical/ or ethics, research/ or exp professional misconduct/ (80281)
15 ((bioethic$ or ethic$) adj3 (standard$ or implicat$ or responsib$ or dilemma$ or problem$)).tw. (14430)
16 or/10-15 (171285)
17 exp Publishing/ (51941)
18 exp publications/ or exp teaching materials/ (217291)
19 publish$.tw. (416175)
20 (publication$ or journal$ or textbook$ or handbook$ or presentation$ or poster$).tw. (777499)
21 or/17-20 (1333943)
22 9 and 16 and 21 (2015)

****

SCOPUS

((TITLE-ABS-KEY(photograph* OR imag*) OR TITLE-ABS-KEY((medical OR clinical OR patient OR dental) W/3 (record* or data)) OR TITLE-ABS-KEY((eye* OR face OR facial) W/3 mask*)) OR TITLE-ABS-KEY((eye* OR face OR facial) W/3 blurr*)) OR TITLE-ABS-KEY(black stripe)) AND (TITLE-ABS-KEY((patient OR written OR verbal OR informed) W/3 consent) OR TITLE-ABS-KEY(patient W/3 (privacy OR confidentiality OR agreement OR anonymity)) OR TITLE-ABS-KEY((bioethic* OR ethic*) W/3 (standard* OR implicat* OR responsib* OR dilemma* OR problem*))) AND (TITLE-ABS-KEY(publish* OR publication* OR journal* OR textbook* OR handbook* OR presentation* OR poster*))
